# Corticosteroid switch from prednisone to dexamethasone in metastatic castration-resistant prostate cancer patients with biochemical progression on abiraterone acetate plus prednisone

**DOI:** 10.1186/s12885-021-08670-2

**Published:** 2021-08-13

**Authors:** Zhenyu Yang, Yuchao Ni, Diwei Zhao, Yijun Zhang, Jun Wang, Lijuan Jiang, Dong Chen, Zhiming Wu, Yanjun Wang, Liru He, Yanxia Shi, Fangjian Zhou, Hao Zeng, Yonghong Li

**Affiliations:** 1 Department of Urology, Sun Yat-sen University Cancer Center, Guangzhou, 510060 China; 2 State Key Laboratory of Oncology in South China, Collaborative Innovation Cencer for Cancer Medicine, Sun Yat-sen University Cancer Center, Guangzhou, 510060 China; 3 Department of Urology, Institute of Urology, West China Hospital, Sichuan University, Chengdu, 610041 China; 4 Department of Pathology, Sun Yat-sen University Cancer Center, Guangzhou, 510060 China; 5 Department of Radiation Oncology, Sun Yat-sen University Cancer Center, Guangzhou, 510060 China; 6 Department of Medical Oncology, Sun Yat-sen University Cancer Center, Guangzhou, 510060 China

**Keywords:** Abiraterone, Castration-resistant prostate cancer, Prednisone, Dexamethasone, Corticosteroid switch

## Abstract

**Background:**

To assess the efficacies and potential predictors of a corticosteroid switch in metastatic castration-resistant prostate cancer (mCRPC) patients with biochemical progression on abiraterone acetate plus prednisone (A + P).

**Methods:**

Patients with mCRPC treated between April 2016 and August 2020, who experienced biochemical progression on A + P and then switched to A plus dexamethasone (D), were retrospectively identified. The primary endpoint was progression-free survival (PFS), and the secondary endpoints were PSA response, overall survival (OS), and safety.

**Results:**

One hundred and thirty consecutive cases were enrolled. The median PFS and OS on A + D were 5.0 and 18.7 months, respectively. The best PSA decline of ≥50% (PSA50) and ≥ 30% (PSA30) were observed in 29.2 and 46.2% patients, respectively. Lower PSA at corticosteroid switch (≤ 20 ng/mL; median PFS, HR 0.63, *p* = 0.019; median OS, HR 0.38, *p* = 0.001) and longer mCRPC-free survival (≥ 18 months; median PFS, HR 0.61, *p* = 0.013; median OS, HR 0.51, *p* = 0.015) were identified as independent prognostic predictors associated with longer PFS and OS. A risk stratification tool was developed to select candidates for corticosteroid switch based on the independent prognostic predictors of PFS and OS.

**Conclusions:**

A corticosteroid switch from prednisone to dexamethasone is effective for mCRPC which progressed on A + P treatment. Patients with lower PSA at corticosteroid switch and/or longer mCRPC-free survival may gain more benefits by the corticosteroid switch.

## Background

Metastatic castration-resistant prostate cancer (mCRPC) is one of the leading causes of death among men worldwide [1]. Although novel therapeutic options were developed for mCRPC, no therapy is curative, and patients with mCRPC have a short overall survival (OS) [2–5].

Abiraterone (A) is a potent inhibitor of CYP17, which blocks gonadal and non-gonadal androgen steroidogenesis [6]. Continuous CYP17 inhibition also blocks adrenal glucocorticoid synthesis and results in elevated adrenocorticotropic hormone levels, which may lead to secondary mineralocorticoid excess characterized by fluid retention, hypertension, hypokalemia and cardiovascular events [7]. Therefore, A is administered in combination with 5 mg prednisone (P) twice daily for mCRPC patients to prevent these side effects [3].

The OS of mCRPC patients is prolonged by the treatment with A plus P (A + P) [3, 8] . However, the survival benefit of ~ 4 months is still limited. Notably, P is not the only concomitant corticosteroid used with A. Dexamethasone (D) 0.5 mg once daily was used in a trial of A reported by Reid et al. [9]. Their study showed a prostate-specific antigen (PSA) decline of ≥50% (PSA50) in 51% of patients, whereas a PSA50 rate of 43% was observed in another trial with 5 mg P twice daily [10]. Moreover, A + P at 5 mg twice daily or D at 0.5 mg once daily was compared in a trial reported by Attard et al. [11]. Therein, a median radiographic progression-free survival (PFS) of 26.6 and 18.5 months were observed in patients treated with D and P, respectively. Interestingly, several studies have reported that the switch from P to D in mCRPC would achieve secondary responses [12–18]. These studies reported PFS of 2.5–11.8 months and PSA50 in 11–55% of patients. The differential responses suggested that not all patients who progressed on A + P could benefit from the corticosteroid switch. However, previous studies with a relatively small cohort (≤ 48 cases) did not distinguish candidates who may have OS benefits from a corticosteroid switch [12–18].

The aim of our study was therefore to assess the efficacies and potential predictors based on PFS and OS of a corticosteroid switch from A to D in mCRPC patients with PSA progression on A + P.

## Materials and methods

### Patients

Data of mCRPC patients with Eastern Cooperative Oncology Group (ECOG) performance status score ≤ 2 subject to a corticosteroid switch from A + P to A + D between April 2016 and August 2020 at West China Hospital (Chengdu, China) and Sun Yat-sen University Cancer Center (Guangzhou, China) was retrospectively analyzed. Report of these data was approved by the institutional review board and ethical committee of the two institutions. However, due to the retrospective nature of this study, ethical committee of West China Hospital and Sun Yat-sen University Cancer Center waived the informed consent for this study. All methods were performed in accordance with the Declaration of Helsinki. Patients that had PSA progression on A + P were switched from P to D. The PSA progression was defined by PCWG3 criteria [19].

### Treatments and outcomes

In this study, the corticosteroid switch from P 5 mg twice daily to D 0.5 mg once daily was performed after PSA progression. Abiraterone 1000 mg once daily was continued. All patients had castrate levels of testosterone < 50 ng/dL. The A + D treatment was maintained until biochemical progression. Biochemical progression was evaluated according to PCWG3 criteria [19]. Biological and Clinical evaluations were performed monthly for the first 3 months, and every 1–3 months thereafter, or any time in case of clinical symptoms. Imaging assessments were performed every 3–6 months, or any time in case of clinical and/or biological progression.

The primary endpoint of this study was PFS, which was defined as the time interval from corticosteroid switch to PSA progression according to PCWG3 criteria [19]. The secondary endpoints of this analysis included OS, PSA response and safety. In this context, OS was defined as the time interval between the corticosteroid switch and death from any causes. PSA response was defined as the best PSA decline of ≥50% (PSA50) and ≥ 30% (PSA30). Any adverse events were evaluated according to the National Cancer Institute Common Terminology Criteria version 5.0.

### Statistical analysis

The continuous variables are expressed as median values, and the categorical variables are presented as frequency (%). Factors predicting PFS and OS were determined by Univariate Cox regression. Those factors with *p* < 0.05 were further tested in multivariate Cox models. Different risk groups were developed according to the multivariate Cox models. The survival curves were generated by the Kaplan-Meier method, and differences were assessed by a log-rank test. All reported *p* values in this study with statistical significance defined as *p* < 0.05 were two-sided. Statistical analyses were carried out with GraphPad Prism version 7 and SPSS version 22.

## Results

### Patient characteristics

One hundred and thirty eligible patients were included in this study; among them, 79 were from one hospital and 51 from another. The clinical characteristics of these patients are shown in Table 1. Eighteen patients received docetaxel therapy after diagnoses with mCRPC. The median age (range) was 73 (50–91) years. The median (range) PSA level at corticosteroid switch was 21.1 (2.2–2893.0) ng/mL.

**Table 1:** Baseline characteristics of patients at the time of corticosteroid switch

Characteristics	No. of patients (%)
Median age (range), years	73 (50–91)
ISUP grading at diagnosis	
≤ 4	43 (33.1)
> 4	75 (57.7)
NA	12 (9.2)
Median time to mCRPC (range), months	13.7 (2.0–168.5)
Chemotherapy before A + P	
Yes	18 (13.8)
No	112 (86.2)
ECOG performance status at corticosteroid switch	
0	76 (58.5)
1, 2	54 (41.5)
Metastasis at corticosteroid switch	
Bone	123 (94.6)
Lymph node	35 (26.9)
Visceral	21 (16.1)
Median PFS of A + P (range), months	6.6 (1.0–48.3)
PSA response to A + P	
PSA decline ≥50%	71 (54.6)
PSA decline ≥30% and < 50%	12 (9.2)
No response	47 (36.1)
Median PSA level (range), ng/mL	
At diagnosis	100.1 (3.7–11,640.0)
At A + P initiation	36.7 (1.2–3126.0)
At corticosteroid switch	21.1 (2.2–2893.0)
PSA nadir after corticosteroid switch	14.0 (0.02–3133.0)
Hemoglobin, g/dL	
≥ 13	39 (30.0)
< 13	62 (47.7)
NA	29 (22.3)
ALP, U/L	
Normal	68 (52.3)
High	27 (20.8)
NA	35 (26.9)
LDH, U/L	
Normal	69 (53.1)
High	24 (18.5)
NA	37 (28.5)

A: abiraterone acetate; ALP: alkaline phosphatase; CRPC: Castration-resistant prostate cancer; D: dexamethasone; ECOG: Eastern Cooperative Oncology Group; ISUP: The International Society of Urological Pathology; LDH: lactate dehydrogenase; NA: not available; P: prednisone; PFS: progression-free survival; PSA: prostate-specific antigen

### PSA and survival outcomes

**Fig. 1:**
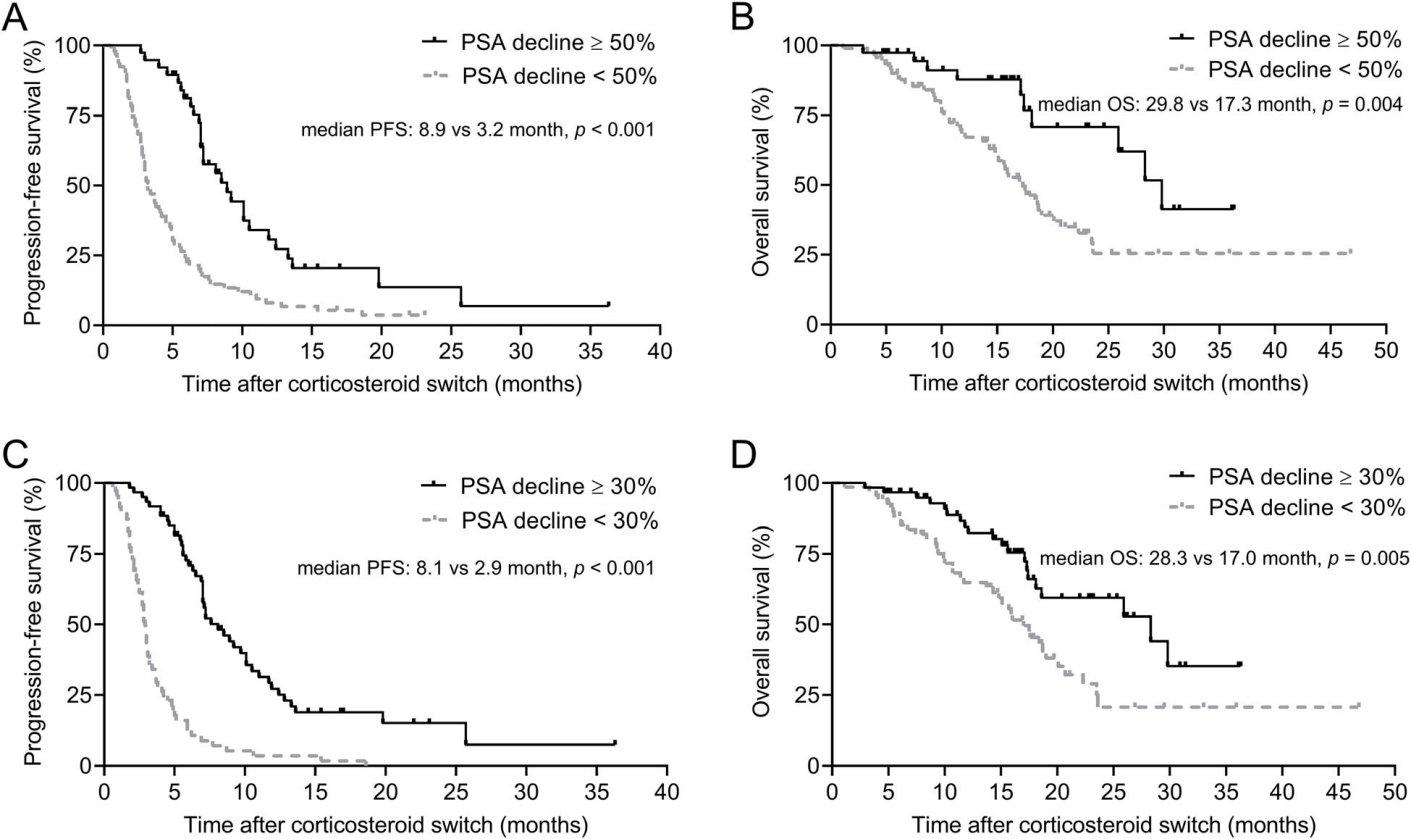
Kaplan-Meier survival curves of PFS (**A**: PSA50; **C**: PSA30) and OS (**B**: PSA50; **D**: PSA30) stratified by PSA response to A + D treatment. PFS: progression-free survival; OS: overall survival

At the time of data cut-off, 20 patients (15.4%) were still responding on A + D; 110 patients (84.6%) had biochemical progression, and 78 (70.9%) of them received further treatments. PSA50 and PSA30 were observed in 38 (29.2%) and 60 patients (46.2%), respectively. The median follow-up was 15.0 months (95% CI, 12.2–15.8). At the last follow-up, 60 patients (46.2%) died from various causes. The observed median PFS of A + D treatment was 5.0 months (95% CI, 3.9–6.1 months), and median OS was 18.7 months (95% CI, 15.7–21.5 months). The median PFS was 8.9 and 3.2 months among patients with and without PSA50 (Fig. 1A), 8.1 and 2.9 months among those with and without PSA30 (Fig. 1C), respectively. The median OS was 29.8 and 17.3 months among patients with and without PSA50 (Fig. 1B), 28.3 and 17.0 months among those with and without PSA30 (Fig. 1D), respectively.

### Univariate and multivariate prognostic analyses

**Fig. 2:**
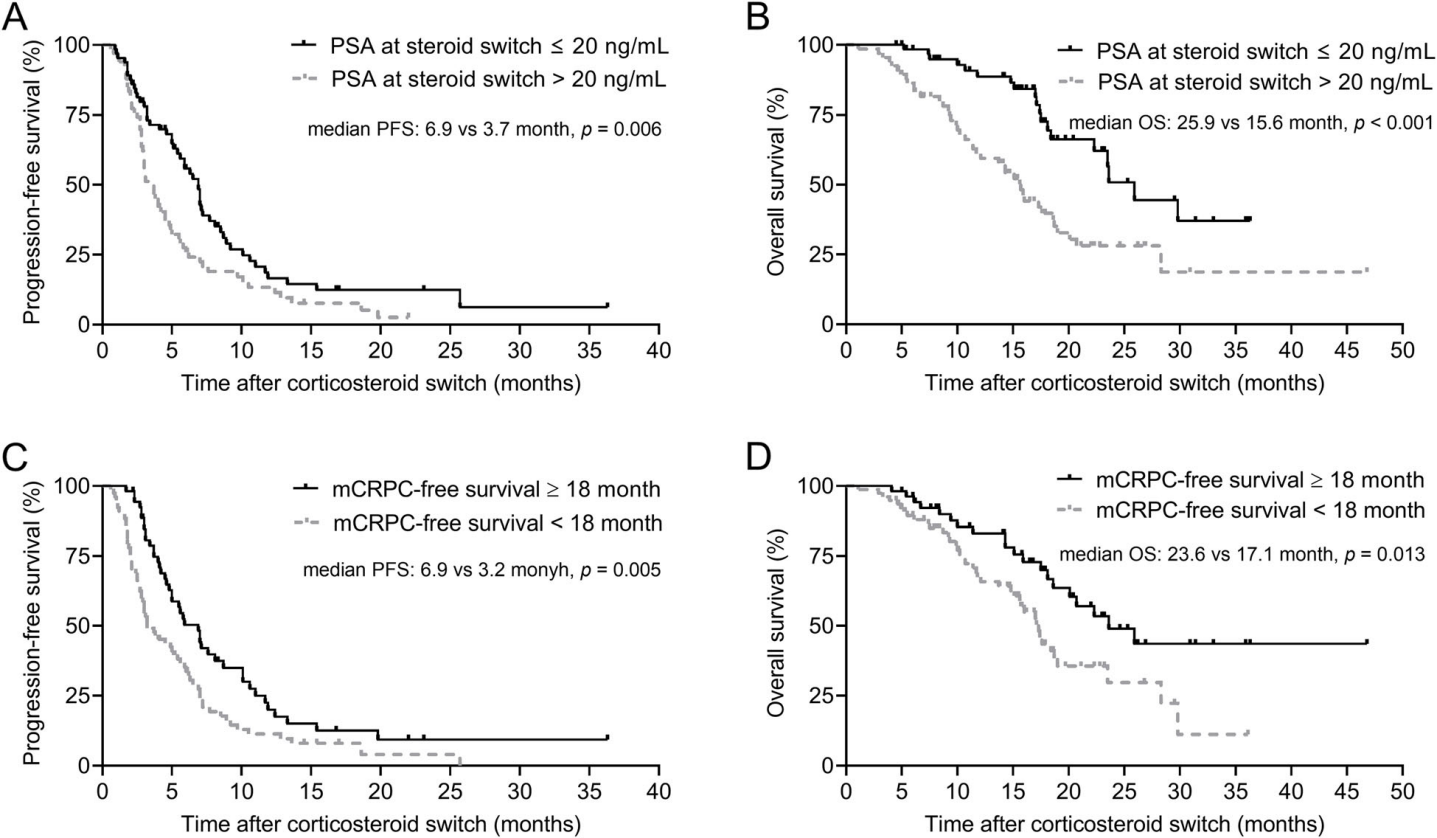
Kaplan-Meier survival curves of PFS and OS stratified by PSA at corticosteroid switch (**A**: PFS; **B**: OS), mCRPC-free survival (**C**: PFS; **D**: OS). mCRPC: metastatic castration-resistant prostate cancer; PFS: progression-free survival; OS: overall survival

The univariate Cox analyses of factors predicting PFS and OS for the corticosteroid switch are shown in Table 2. Due to lack of data for some patients, ALP, LDH and hemoglobin were not analyzed. Among all the tested factors, two factors were found associated with both longer PFS and OS, including lower PSA level (≤ 20 ng/mL) at corticosteroid switch and longer mCRPC-free survival (≥ 18 months). Patients with better ECOG performance status score (0) also had longer OS. In multivariate analyses, PSA level (≤ 20 ng/mL) at corticosteroid switch and mCRPC-free survival (≥ 18 months) were identified as independent factors in predicting both PFS and OS (Table 3). PFS curves are shown in Fig. 2A and C, while OS curves are shown in Fig. 2B and D.

**Table 2:** Univariate Cox analyses predicting PFS and OS of corticosteroid switch

	N	PFS, months			OS, months		
		Median (95%CI)	HR (95%CI)	*p* value	Median (95%CI)	HR (95%CI)	*p* value
Age at corticosteroid switch, years						1.17 (0.70–1.98)	0.563
≥ 70	81	5.5 (4.4–6.6)	0.75 (0.51–1.10)	0.139	18.1 (16.7–19.5)		
< 70	49	3.2 (0.7–5.7)			23.5 (13.5–33.5)		
ISUP grading at diagnosis						0.89 (0.51–1.55)	0.670
> 4	75	4.8 (3.5–6.1)	1.01 (0.67–1.53)	0.946	20.1 (14.3–25.9)		
≤ 4	43	5.5 (2.9–8.1)			17.3 (13.4–21.2)		
ECOG at corticosteroid switch						0.58 (0.34–0.97)	0.037
0	76	5.6 (4.2–7.0)	0.75 (0.51–1.10)	0.140	23.5 (17.3–29.7)		
≥ 1	54	3.7 (2.2–5.2)			17.4 (13.8–21.0)		
PSA at diagnosis, ng/mL						1.01 (0.58–176)	0.978
≥ 100	76	4.8 (3.7–5.9)	1.09 (0.71–1.65)	0.703	19.0 (14.2–23.8)		
< 100	41	5.9 (5.0–6.8)			18.1 (14.2–22.0)		
PSA at corticosteroid switch, ng/mL						0.37 (0.21–0.54)	< 0.001
≤ 20	64	6.9 (5.8–8.0)	0.60 (0.41–0.87)	0.007	25.9 (21.7–30.1)		
> 20	66	3.7 (2.8–4.6)			15.6 (13.3–17.9)		
mCRPC-free survival, months						0.51 (0.30–0.87)	0.014
≥ 18	53	6.9 (5.2–8.6)	0.58 (0.40–0.85)	0.005	23.6 (17.7–29.5)		
< 18	77	3.2 (2.0–4.4)			17.1 (15.1–19.1)		
Time to progression on A + P, months						0.98 (0.58–1.65)	0.930
≥ 8	50	6.2 (4.5–7.9)	0.75 (0.51–1.10)	0.144	17.5 (15.3–19.7)		
< 8	80	4.2 (2.6–5.8)			19.0 (14.9–23.1)		
PSA response on A + P						1.46 (0.86–2.46)	0.161
Decline ≥50%	72	4.9 (3.8–6.0)	1.11 (0.75–1.62)	0.608	17.4 (16.2–18.7)		
Decline < 50%	58	5.4 (3.6–7.2)			28.3 (14.9–41.7)		

A: abiraterone acetate; CI: confidence interval; ECOG: Eastern Cooperative Oncology Group; HR: hazard ratio; ISUP: The International Society of Urological Pathology; mCRPC: metastatic castration-resistant prostate cancer; OS: overall survival; P: prednisone; PFS: progression-free survival; PSA: prostate-specific antigen

**Table 3:** Multivariate Cox analyses predicting PFS and OS of corticosteroid switch

	N	PFS		OS	
		HR (95%CI)	*p* value	HR (95%CI)	*p* value
ECOG at corticosteroid switch				0.58 (0.35–0.97)	0.040
0	76				
≥ 1	54				
PSA at corticosteroid switch, ng/mL		0.63 (0.43–0.93)	0.019	0.38 (0.22–0.66)	0.001
≤ 20	64				
> 20	66				
mCRPC-free survival, months		0.61 (0.41–0.90)	0.013	0.51 (0.30–0.88)	0.015
≥ 18	53				
< 18	77				

CI: confidence interval; D: dexamethasone; HR: hazard ratio; mCRPC: metastatic castration-resistant prostate cancer; OS: overall survival; PFS: progression-free survival; PSA: prostate-specific antigen

### Prognostic risk stratification

**Fig. 3:**
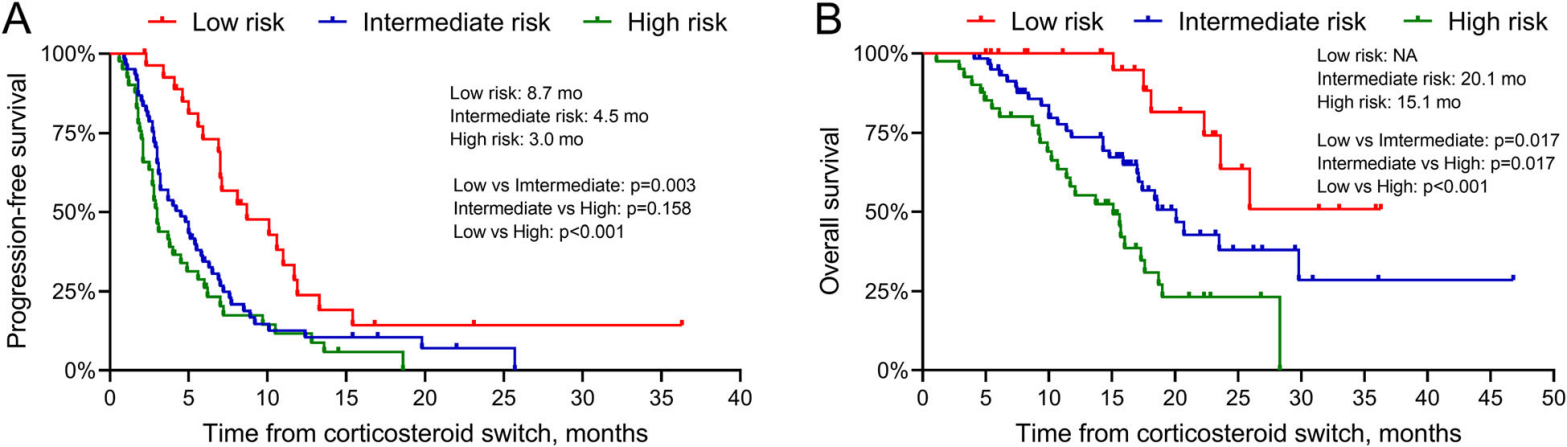
Progression-free survival (**A**) and overall survival (**B**) in different risk groups

Because ECOG performance status itself may affect survival, three risk groups were further defined based on only two factors (PSA at corticosteroid switch and mCRPC-free survival) which can predict both PFS and OS independently in multivariate Cox analysis: low-risk group (*n* = 28), with two favorable prognostic predictors (PSA at corticosteroid switch ≤20 ng/mL and mCRPC-free survival ≥18 months); intermediate-risk group (*n* = 61), with one unfavorable prognostic predictor (PSA at corticosteroid switch ≤20 ng/mL and mCRPC-free survival < 18 months; PSA at corticosteroid switch > 20 ng/mL and mCRPC-free survival ≥18 months); and high-risk group (*n* = 41), with two unfavorable prognostic predictors (PSA at corticosteroid switch > 20 ng/mL and CRPC-free survival < 18 months). Univariate Cox analyses indicate that patients in the intermediate-risk and high-risk groups featured 2.01- and 2.73-fold risk of progression, and 2.83- and 5.62-fold risk of death, respectively (Table 4), compared with patients with low-risk. Survival curves of PFS and OS for these groups are shown in Fig. 3. The PFS of low-risk group was significantly longer than intermediate-risk group (median 8.7 vs 4.5 months, *p* = 0.003) and high-risk group (median 8.7 vs 3.0 months, *p* < 0.001), whereas the PFS between intermediate-risk and high-risk were not significantly different (median 4.5 vs 3.0 months, *p* = 0.160). This risk stratification was able to separate the OS (median NA vs 20.1 vs 15.1 months in low-, intermediate- and high-risk groups, respectively, *p* < 0.05) of all groups. Rates of PSA50 were 35.7, 32.7 and 19.5% for low-, intermediate- and high-risk groups, respectively.

**Table 4:** Results of univariate Cox analyses in the prognostic risk stratification

Risk group	N	PFS			OS		
		Events	HR (95% CI)	*p* value	Events	HR (95% CI)	*p* value
Low	28	20	1		6	1	
Intermediate	61	52	2.01 (1.19–1.37)	0.009	27	2.83 (1.16–6.87)	0.022
High	41	38	2.73 (1.58–4.72)	< 0.001	27	5.62 (2.30–13.78)	< 0.001

CI: confidence interval; HR: hazard ratio; OS: overall survival; PFS: progression-free survival

### Safety

A + D treatment was well-tolerated after the corticosteroid switch for all patients in this study. No adverse events grade 3/4 were observed, and no dose reduction of D was required. No treatment-related death happened. Fluid retention (*n* = 6, 4.6%), fatigue (*n* = 4, 3.1%), hypokalaemia (*n* = 3, 2.3%), hypertension (*n* = 3, 2.3%), and hyperglycaemia (*n* = 2, 1.5%) were the most common adverse events. All of these adverse events were grade 1/2.

## Discussion

A switch of corticosteroid from 5 mg P twice daily to 0.5 mg D once daily after progression on A + P was able to reverse biochemical resistance, as reported by several studies [12–18]. The corticosteroid switch could prolong PFS of 2.5–11.8 months, and result in PSA50 and PSA30 of 11–55% and 40–45%, respectively. In the present study, PSA50 and PSA30 were observed in 28.5 and 46.2% of patients, respectively; the median PFS and OS were 5.0 and 18.7 months, respectively, after the corticosteroid switch. These results further support the benefits from this corticosteroid switch. Long-term use of dexamethasone may result in some adverse events. Considering that dexamethasone is inexpensive and relative short-term use is safe, patients can easily and safely obtain A + D treatment after progression on A + P. This can be helpful especially for patients who cannot afford other effective sequential therapies, or when few sequential agents are available.

Although all previous studies have attributed a positive clinical value to corticosteroid switch, its efficacy appears not completely consistent. For example, PSA50 ranged from 11 to 55%, and median PFS was 2.5–11.8 months in these studies [12–18]. In our cohort, no PSA decline happened in 33.1% patients, while 20 patients presented PFS ≤ 2 months. These results indicate that not all patients show a satisfactory response to corticosteroid switch, and emphasizes the importance of candidate selection for corticosteroid switch in clinical practice. Fenioux et al. [12] developed a prognostic score to distinguish patients with longer PFS for A + D based on independent favorable prognostic factors predicting PFS. However, the study involved only a small cohort (48 patients) with 14 and 9 patients in low- and high-risk groups, respectively. Besides, whether longer PFS of patients corresponded with OS benefits was not clear, since OS was not reported therein.

In this study, two factors favorable for both PFS and OS of corticosteroid switch were considered: lower PSA (≤ 20 ng/mL) at corticosteroid switch and longer mCRPC-free survival (**≥** 18 months). Longer mCRPC-free survival, which is associated with longer PFS after corticosteroid switch, was shown by previous studies, as reported by Fenioux et al. [12] and Rivero-Belenchón et al. [16]. Furthermore, lower PSA at corticosteroid switch indicated longer PFS after corticosteroid switch [12]. Fenioux et al. [12] reported that short PFS for A + P is a favorable factor predicting PFS after corticosteroid switch; however, Romero-Laorden et al. [14] had contrasting results. Interestingly, PFS for A + P was not an independent factor predicting the effect of the corticosteroid switch in our study and the study reported by Rivero-Belenchón et al. [16]. Further prospective studies with more enrolled patients are therefore needed to clarify the association between PFS for A + P and the efficacy of corticosteroid switch.

A risk stratification tool was developed based on the two independent prognostic predictors of PFS and OS in our study, including PSA at corticosteroid switch and mCRPC-free survival, to select candidates for the corticosteroid switch. Although the PFS was not different significantly between patients in intermediate- and high-risk groups, patients in all risk groups were well-separated based on OS. Furthermore, PSA50 was better in low- (35.7%) and intermediate-risk (32.7%) groups, as compared with high-risk group (19.5%). Therefore, patients with low- and intermediate-risk may be appropriate candidates to receive a corticosteroid switch after biochemical progression on A + P treatment, as they may gain more survival benefits from the switch. On the other hand, patients with high-risk may be not suitable to receive the switch due to poor responses and low survival benefits.

To date, the exact mechanism underlying the corticosteroid switch to reverse A + P resistance remains unclear. Several hypotheses have been proposed to explain this effect [20]. First, the activation of glucocorticoid receptor (GR) leads to resistance. Dexamethasone 0.5 mg has lower equivalent glucocorticoid activity when compared with prednisolone 10 mg, thus fewer GRs are activated by Dexamethasone [21]. Second, AR point mutations of AR can be activated by prednisone, rather than by dexamethasone [22]. Third, pharmacodynamic and pharmacokinetic differences between prednisone and dexamethasone lead to higher efficacy of dexamethasone in suppressing the adrenocorticotropic hormone [23]. Fourth, resistance due to the activation of mineralocorticoid receptors, for which dexamethasone has lower affinity [24]. Nevertheless, none of these hypotheses have been fully verified, thus further research is needed to uncover the mechanism of the corticosteroid switch.

The activity of D itself may be involved in the antitumor effect; it has been shown that D can suppress lymphangiogenesis in prostate cancer [25]. A was administered without steroids in a phase I/II study, and D 0.5 mg once daily was added to A after PSA progression, which treatment resulted in PSA50 in 33% of patients [26]. In another phase I/II trail that prior to the clinical use of enzalutamide and A in mCRPC patients, D monotherapy was found more effective than P [27]. These results may explain why PFS for A + P treatment is not a predictive factor of the efficacy of corticosteroid switch. Whether it is A + D treatment or D monotherapy that leads to the antitumor effect after progression on A + P treatment needs to be elucidated by further research.

The limitations of current study include the retrospective nature of this study, which may lead to potential bias. Moreover, a control group that D monotherapy was administered when corticosteroid switch was carried out was also lacking. Thus, the possibility of the antitumor effect being related to D monotherapy cannot be excluded. A + D treatment was terminated due to PSA progression in most patients, hence A + D treatment length was less than three months for some patients. Furthermore, LDH, Hb and ALP were not included in univariate and multivariate analyses due to lack of data, which may be significant prognostic factors; and only patients with ECOG≤2 were included, this could have impact on OS. Finally, sequential treatments after progression on A + D could influence OS, and this was not analyzed in this study.

## Conclusions

The present study adds to evidence that the corticosteroid switch from prednisone to dexamethasone is a feasible and effective alternative for mCRPC patients who failed from A + P treatment. Patients with lower PSA at corticosteroid switch and/or previous longer mCRPC-free survival may gain more treatment benefits after this type of corticosteroid switch.

## Data Availability

The datasets used and/or analyzed during the current study are available from the corresponding author on reasonable request.

## Abbreviations

A: abiraterone acetate
D: dexamethasone
ECOG: Eastern Cooperative Oncology Group
mCRPC: metastatic castration-resistant prostate cancer
OS: overall survival
P: prednisone
PCWG: Prostate Cancer Working Group
PFS: progression-free survival
PSA: prostate specific antigen
